# Asymmetric wall-stress heterogeneity defines a stretch-activated arrhythmogenic substrate beyond ejection fraction in dilated cardiomyopathy: an in silico study

**DOI:** 10.3389/fphys.2026.1884294

**Published:** 2026-07-22

**Authors:** Arnav Amit

**Affiliations:** Independent Researcher, Los Angeles, CA, United States

**Keywords:** arrhythmia, computational cardiology, diastolic depolarization, dilated cardiomyopathy, ejection fraction, repolarization heterogeneity, stretch-activated channels, wall stress

## Abstract

In dilated cardiomyopathy (DCM), sudden cardiac death from ventricular arrhythmia occurs unpredictably among patients with similar ejection fraction (EF), and EF performs poorly as an individual risk predictor. EF is a cavity-volume ratio and is, by construction, blind to the spatial distribution of wall thinning. I hypothesized that the pattern of remodeling, specifically asymmetric septal versus lateral wall thinning, produces spatially heterogeneous diastolic wall stress and stretch, which through stretch-activated channel (SAC) activation creates an arrhythmogenic substrate that EF cannot encode. I developed a three-component in silico model coupling a parametric prolate-ellipsoid left ventricular geometry, regional biaxial (Laplace) wall stress, and the O’Hara-Rudy 2011 human ventricular action potential model with an ohmic SAC current engaged during diastole. Across five severity by five asymmetry levels, ejection fraction was computed and depended on severity alone, not on asymmetry. At matched EF, asymmetric remodeling produced a regional gradient of end-diastolic stretch that uniform dilation did not: at severe asymmetric DCM the thinned septum was depolarized by 2.25 mV relative to baseline, with 1.32 mV of regional resting-potential dispersion and 2.19 ms of action potential duration (APD) dispersion, against essentially none for uniform dilation at the same EF. The substrate rose approximately linearly with the septal/lateral wall-stress gradient, which equals the lateral-to-septal wall-thickness ratio measurable on a standard echocardiogram (slope 1.81 mV per unit gradient), and saturated at the sarcomere stretch ceiling. This in silico study quantifies a known mechanism and converts it into a specific, falsifiable prediction: an echo-derived wall-thickness ratio indexes an arrhythmic substrate orthogonal to EF, motivating retrospective and prospective clinical validation.

## 1 Introduction

Dilated cardiomyopathy (DCM) affects approximately 1 in 250 individuals and is a leading cause of sudden cardiac death from ventricular arrhythmia (Weintraub et al., 2017). Guidelines recommend implantable cardioverter-defibrillator implantation primarily on the basis of a left ventricular ejection fraction (LVEF) below 35% (Priori et al., 2015), yet this threshold performs poorly as an individual predictor. The majority of sudden cardiac death events in DCM occur in patients with LVEF above 35%, and many patients with severely reduced EF never experience life-threatening arrhythmia. This indicates that EF captures the severity of mechanical dysfunction but misses the specific substrate that makes some DCM ventricles arrhythmogenic.

There is a structural reason EF might miss this substrate. EF is a ratio of cavity volumes, and the spatial distribution of wall thinning is not one of its inputs. EF can be computed without any knowledge of how thinning is distributed between the septum and the lateral wall. Any feature that lives in that distribution is therefore invisible to EF by definition, not merely by limited sensitivity.

The link between dilation and arrhythmia is incompletely understood but involves mechanoelectric feedback. Cardiomyocytes express stretch-activated ion channels whose conductance rises with membrane deformation, and these channels are engaged predominantly during diastolic filling, when lengthened myocytes sit at resting potential and carry an inward, depolarizing non-selective cation current (Kohl et al., 1999; Peyronnet et al., 2016; Kamkin et al., 2000). In the dilated ventricle regional wall stress is elevated and spatially nonuniform, so regions stretch by different amounts depending on local thickness and curvature. Heterogeneous diastolic stretch produces heterogeneous SAC engagement, which shifts the resting membrane potential and the action potential differently across regions. Spatial dispersion of resting potential and of repolarization is an established arrhythmogenic substrate (Glukhov et al., 2010; Coronel et al., 2009).

Existing work has examined global stretch in single cells or uniformly stretched preparations, and clinical metrics such as EF and global strain are scalar summaries that collapse spatial information. The question this study addresses is narrower and more specific: whether the geometric pattern of remodeling, the degree of asymmetric septal versus lateral wall thinning, determines an arrhythmic substrate at a fixed EF, and whether that substrate is indexed by a quantity already measured on a standard echocardiogram.

A deterministic in silico model does not discover facts about real hearts; it quantifies a hypothesis and generates a prediction. The individual links in the chain above are each established, so their composition is expected rather than surprising. The contribution sought here is therefore threefold: to quantify how much substrate a given wall-thickness asymmetry produces, to show that this substrate is orthogonal to EF, and to convert the qualitative mechanism into a specific, echo-computable, falsifiable prediction for clinical testing.

## 2 Materials and methods

### 2.1 Overview

The model has three coupled components: a parametric prolate-ellipsoid LV geometry, regional biaxial wall stress from thin-wall membrane (Laplace) theory, and a human ventricular electrophysiology model with a stretch-activated current engaged in diastole. All code was implemented in Python 3.11 using myokit with the SUNDIALS CVODE solver for electrophysiology and numpy for geometry and analysis. No animal or human subjects were used. Model code and generated data are available at https://github.com/arnava25/dcm-arrhythmia-model.

### 2.2 Left ventricular geometry

**Figure 1:**
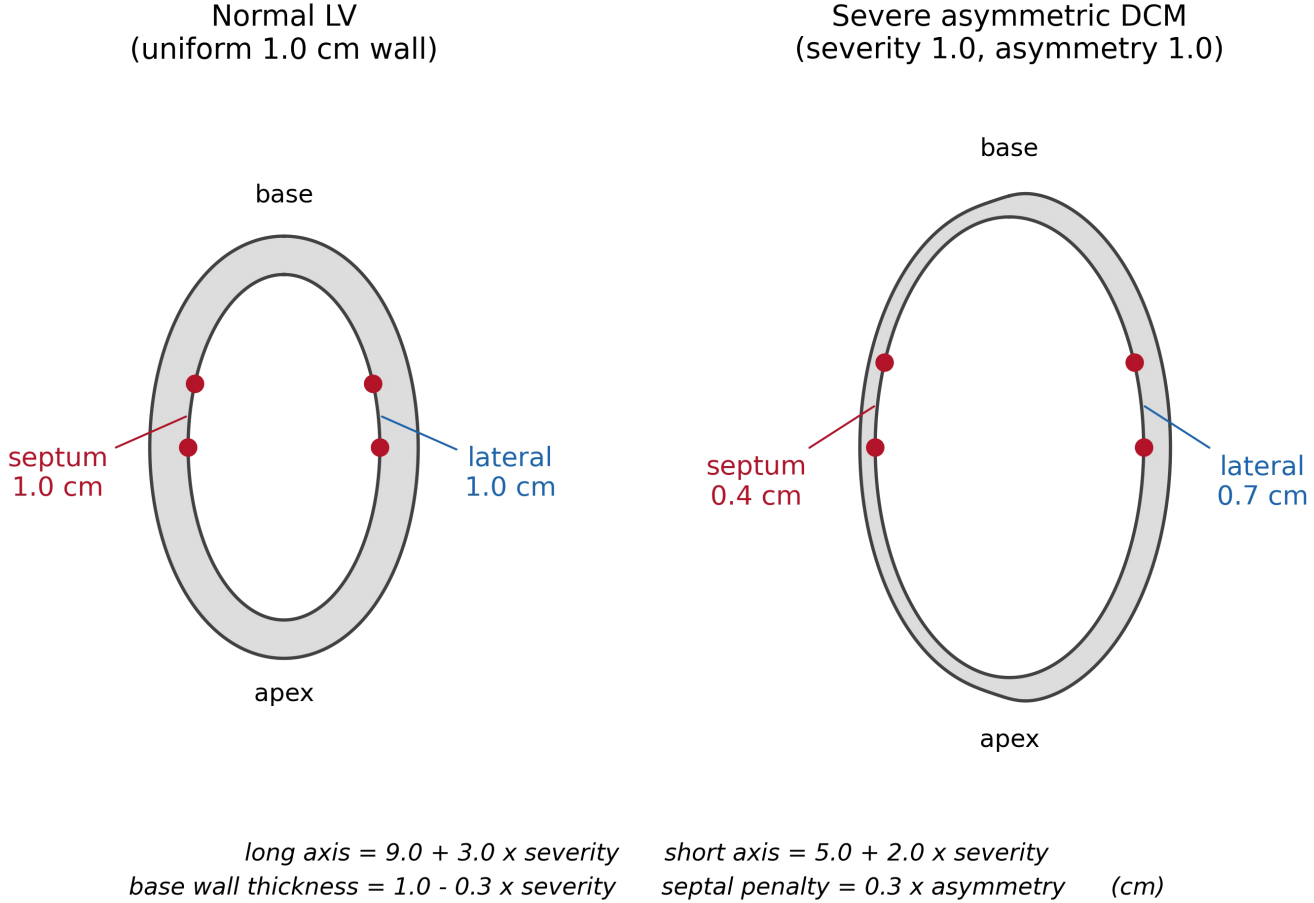
Geometry schematic. Long-axis cross-section of the endocardial and epicardial contours (wall shaded) for the normal LV with a uniform 1.0 cm wall (left) and for severe asymmetric DCM (right), in which the septum is thinned to 0.4 cm while the lateral wall is preserved at 0.7 cm and the chamber is dilated. Red markers indicate the four non-apical regions, placed at matched septal and lateral longitudinal levels. The parameter equations relating severity and asymmetry to the geometry are shown beneath the panels.


The left ventricle was modeled as a prolate ellipsoid with endocardial polar semi-axis a and equatorial semi-axis b. Five regions were defined: basal septal, mid septal, apical, mid lateral, and basal lateral. Septal and lateral regions were placed at matched longitudinal levels, that is at the same parametric angle from the apex, so the surface is axisymmetric and any septal versus lateral stress difference arises only from differential wall thinning rather than from the parametrization. The geometry is given by explicit parameter equations (**Figure 1**):

$$long\;axis\;=\;9.0\;+\;3.0\;x\;severity\;\left(cm\right)\;short\;axis\;=\;5.0\;+\;2.0\;x\;severity\;\left(cm\right)$$

$$base\;wall\;thickness\;=\;1.0\;-\;0.3\;x\;severity\;\left(cm\right)\;septal\;penalty\;=\;0.3\;x\;asymmetry\;\left(cm\right)$$

The septal penalty is subtracted from the two septal regions. The two dimensionless indices, severity and asymmetry, each range from 0 to 1 and are defined as independent controls, so that global dilation and the septal-versus-lateral distribution of thinning can be varied separately; this independence, not anatomical fidelity, is the purpose of the parameterization. The linear coefficients are not fitted to a patient cohort. They are an idealization whose endpoints are anchored to published ranges: at severity 0 the geometry is a 9.0 by 5.0 cm chamber with a uniform 1.0 cm wall, within normal reference ranges (Grossman et al., 1975); at severity 1 the chamber dilates to 12.0 by 7.0 cm with global wall thinning, and at asymmetry 1 the septum thins to 0.4 cm against a 0.7 cm lateral wall, spanning the severe end of the dilation and wall-thinning ranges reported in DCM (Weintraub et al., 2017). Intermediate values interpolate linearly between these anchored endpoints. The model is therefore a controlled idealization for isolating the effect of remodeling pattern, not a morphometric reconstruction of an individual ventricle, a point noted again among the limitations.

### 2.3 Regional wall stress

For the prolate ellipsoid the two principal radii of curvature at each region, the meridional R_m and the transverse R_t, were computed as.


$$Rm=\frac{D^{3}}{ab}\;\frac{R_{t}}{b}\;\frac{D}{a}\text{ with }D=\sqrt{a^{2}\sin^{2}\left(\varphi \right)+b^{2}\cos^{2}\left(\varphi \right)}$$

where phi is the parametric angle from the apex. The two principal thin-wall membrane stresses follow from the membrane equilibrium of a shell of revolution:


$$\sigma_{\text{meridional}}=\frac{PR_{t}}{2h}\;\sigma_{\text{circumferential}}=\frac{PR_{t}}{h}\left(1-\frac{R_{t}}{2R_{m}}\right)$$

where P is cavity pressure and h is regional wall thickness. I report the circumferential (hoop) component, the convention in echocardiographic and cardiac magnetic resonance wall-stress studies (Mirsky, 1969); the meridional component is also computed. This replaces the single-component spherical Laplace law, which is not valid for a prolate geometry that has two distinct principal stresses. Wall stress heterogeneity was quantified as the coefficient of variation of circumferential stress across the four non-apical regions; the apex was excluded because it lies on the axis of symmetry and has no septal or lateral identity. The septal/lateral gradient was defined as mean septal divided by mean lateral circumferential stress. Because septal and lateral regions sit at matched levels, this gradient reduces exactly to the lateral-to-septal wall-thickness ratio, independent of curvature, pressure, and the choice of stress component, and is therefore directly readable from echocardiographic wall-thickness measurements. Both the gradient and the coefficient of variation are ratios and are invariant to the absolute pressure.

LV systolic pressure was set to 120 mmHg as a normotensive reference and end-diastolic pressure to 8 mmHg; the end-diastolic pressure is the operative pressure for diastolic stretch (Section 2.5). Thin-wall membrane stress diverges from finite-element fiber stress, which is acknowledged as a limitation.

### 2.4 Ejection fraction and strain

The semi-axes a and b describe the endocardial cavity surface, so the cavity volume is V = (4/3) pi a b_2_, which gives 118 mL for the normal geometry. Ejection fraction was computed as EF = (EDV - ESV)/EDV with stroke volume held fixed at the value that gives the normal heart an EF of 0.60, applied to every geometry. EF therefore falls as the chamber dilates and depends only on severity, not on asymmetry, because asymmetry redistributes wall thickness without changing the cavity. Global strain was reported as the implied isotropic linear shortening, 1 - (1 - EF)^(1/3)^. EF in an idealized ellipsoid is geometry-confounded, and a normal *in vivo* EF of 60% requires helical fiber architecture that an idealized ellipsoid does not have (Stokke et al., 2017; Triposkiadis et al., 2018); the EF here is a geometric calibration, not a fiber-mechanics derivation, and is used to control for severity, not to model contraction.

### 2.5 Diastolic stretch

Stretch-activated channels are engaged during diastolic filling rather than at peak systole (Peyronnet et al., 2016), so regional fiber stretch was evaluated at end diastole. Stretch is defined in the usual way as the ratio of deformed to reference length, λ = L / L_ref_, with L_ref_ the sarcomere length at the normal end-diastolic loaded state, so that λ = 1.0 denotes normal diastolic loading and λ > 1 denotes lengthening beyond it.

Regional stretch is driven by regional end-diastolic circumferential wall stress, which by the membrane relations of Section 2.3 depends on both local wall thickness and local curvature. A region under higher end-diastolic stress lengthens further; through a linear first-order passive compliance the excess stretch above the reference is proportional to the excess end-diastolic stress, capped at the top of the measured sarcomere working range,


$$\lambda_{\text{max}}=\frac{2.30}{1.85}=1.24$$

taking the working range as approximately 1.9 to 2.3 micrometers relative to a slack length of 1.85 micrometers (Sequeira et al., 2023).

**Figure 2:**
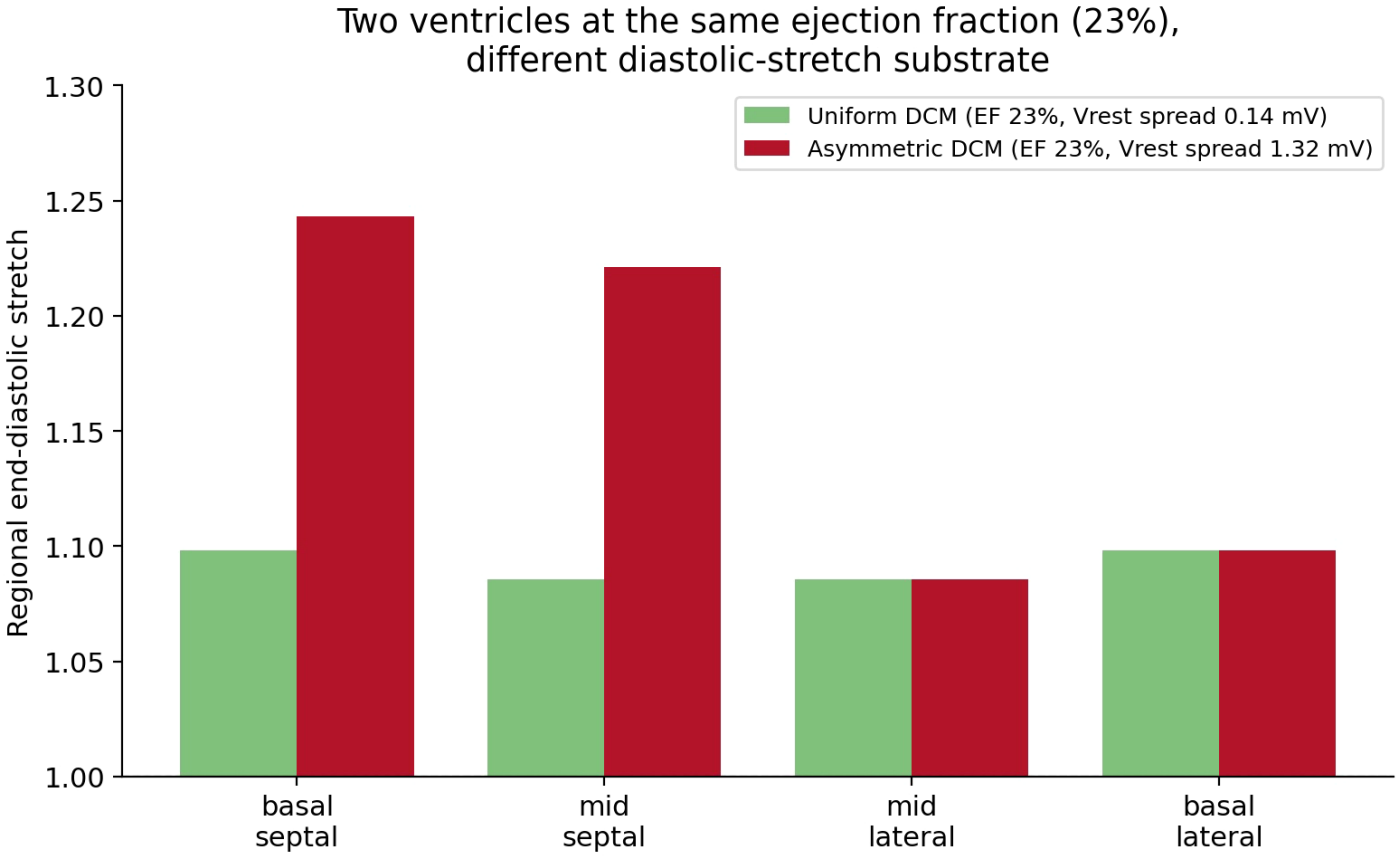
Two ventricles at the same ejection fraction, different substrate. Regional end-diastolic stretch for uniformly dilated DCM and asymmetrically remodeled DCM, both at severity 1.0 and both at an ejection fraction of 23%. Uniform dilation stretches all regions equally; asymmetric remodeling stretches the thinned septal regions further while the lateral wall is unchanged, opening the septal-to-lateral gradient that drives the substrate (resting-potential spread 0.14 mV uniform versus 1.32 mV asymmetric).

This makes the dependence on remodeling pattern explicit. Thinner walls carry higher stress and therefore stretch more, but under uniform dilation every region is thinned by the same amount, so the thickness contribution is equal across regions and only the mild physiological base-to-apex variation in curvature remains; stretch is then nearly homogeneous. Under asymmetric remodeling the septum is thinned more than the lateral wall, so septal end-diastolic stress and stretch exceed lateral, opening the septal-to-lateral stretch gradient that drives the substrate (**Figure 2**). The spatial pattern of stretch is therefore set by geometry alone and is independent of the compliance calibration, which fixes only the absolute magnitude.

The reference is the loaded normal diastolic state rather than the unloaded stress-free configuration, so residual stress is neglected; the passive stress-to-strain relation is linearized; and end-diastolic pressure is held at a fixed baseline. These are acknowledged as limitations.

### 2.6 Electrophysiology and stretch-activated current

Single-cell electrophysiology used the O’Hara-Rudy 2011 human ventricular action potential model (epicardial cell type), comprising 41 state variables, integrated with myokit and CVODE. A stretch-activated current was added to the membrane voltage equation as I_SAC_ = g_SAC_ (V - E_SAC_), with E_SAC = -10 mV and g_SAC rising linearly with stretch from a threshold of 1.0 to a maximum at a stretch of 1.2, capped above. The default maximum conductance was 0.003 nS/pF. These parameters are drawn from the ventricular-myocyte SAC literature (Craelius et al., 1988; Kamkin et al., 2000; Peyronnet et al., 2016); the whole-cell reversal potential spans roughly -90 to -10 mV depending on the relative expression of K-selective and non-selective channels. With E_SAC = -10 mV the current is inward at rest, producing diastolic depolarization, and prolongs APD with stretch, consistent with non-selective-cation-dominant preparations. The direction of the APD change is preparation-dependent, and the arrhythmogenic quantity is the regional spread of repolarization and resting potential, not its sign. The conductance set by diastolic stretch is applied as a constant through the beat, a quasi-static approximation that is acknowledged as a limitation.

Each cell was paced for a fixed 600 beats at 1 Hz, identical across all regions, so that the slow drift of the O’Hara-Rudy model is common to all regions and cancels in the region-to-region dispersion. The beat-to-beat APD90 drift at 600 beats was below 0.012 ms, far below the inter-region dispersion measured. The baseline steady-state APD90 was 228.6 ms. Two readouts were extracted per region: the resting (maximum diastolic) membrane potential, the primary substrate measure, and APD at 90% repolarization, reported as a secondary measure. The substrate was quantified as the range and coefficient of variation across regions and as the diastolic depolarization of the most-stretched region relative to the no-SAC baseline.

### 2.7 Parameter sweep

A factorial sweep was run across five severity levels (0, 0.25, 0.5, 0.75, 1.0) and five asymmetry levels (0, 0.25, 0.5, 0.75, 1.0), giving 25 configurations. Severity 0 is the common normal reference, and asymmetry has no effect in the absence of thinning, leaving 20 distinct DCM geometries. EF, wall stress heterogeneity, the septal/lateral gradient, and both electrophysiological readouts were computed for each.

### 2.8 Sensitivity and validation

Robustness to SAC parameter uncertainty was assessed by varying the maximum conductance across its published range and the reversal potential across its physiological span. Because the spatial stretch pattern is fixed by geometry and the SAC scales it monotonically, the separation between asymmetric and uniform geometries persists across the conductance range, and the reversal potential shifts all regions equally and does not change the relative dispersion. The baseline action potential is validated against human data in its source publication (O'Hara et al., 2011); the added SAC current was validated against preparation-matched experimental data. Homogeneous axial stretch of isolated ventricular myocytes, the preparation the single-cell model corresponds to, produces small late-repolarization changes, on the order of a 7% APD prolongation with a resting depolarization of about 2 mV (Isenberg et al., 2003); local stretch with a shear component raises the late-APD change to roughly 20% (Kamkin et al., 2000); and intact loaded preparations show larger prolongation still, up to +28 ms (APD80–179 to 207 ms, +15.6%) at approximately 4% epicardial strain in the rabbit (Sung et al., 2003). The model is benchmarked against these named values in the single-cell validation (Section 3.5).

## 3 Results

### 3.1 Ejection fraction is controlled by severity, not pattern

**Figure 3:**
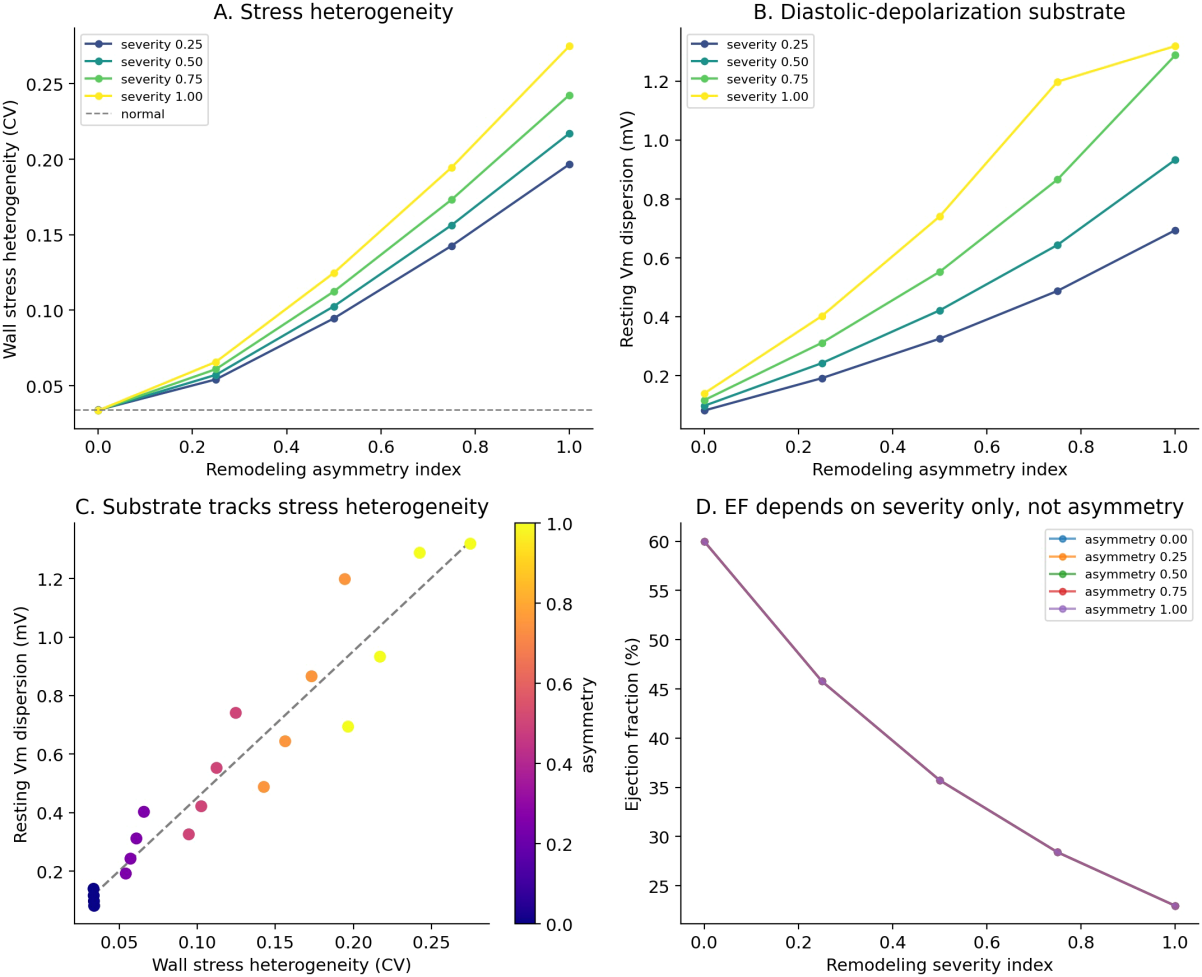
Parameter-space results across the 25 geometries. **(A)** Wall stress heterogeneity (coefficient of variation) rises with asymmetry and is amplified at higher severity; uniform dilation stays at the normal value. **(B)** Regional resting membrane potential dispersion, the diastolic-depolarization substrate, follows the same pattern. **(C)** The substrate tracks wall stress heterogeneity; the relationship is a deterministic consequence of the model and is shown as a relationship, not as an inferential statistic. **(D)** Ejection fraction falls with severity but is identical across all asymmetry levels, so the five asymmetry curves coincide.

EF computed from the geometry was 60% for the normal heart and fell to 23% at maximum severity, and at every severity it was identical across all asymmetry levels (**Figure 3D**). Asymmetry redistributes wall thickness without changing the cavity, so it does not change EF in the model. This makes EF a controlled variable in the comparison that follows: differences in substrate between uniform and asymmetric geometries at a fixed severity occur at an identical EF.

### 3.2 Asymmetry, not dilation, creates wall-stress heterogeneity

In the normal heart and in uniformly dilated DCM the septal/lateral gradient was exactly 1.0 and the coefficient of variation of stress was 0.034, reflecting a near-uniform distribution with only a physiological base-to-apex variation. Uniform dilation did not raise the gradient at any severity. Asymmetric remodeling raised both measures: at severe asymmetric DCM the gradient reached 1.75, equal to the 0.7 to 0.4 lateral-to-septal thickness ratio, and the coefficient of variation reached 0.275. Stress heterogeneity increased monotonically with asymmetry and was amplified at higher severity (**Figure 3A**).

### 3.3 Asymmetry creates a diastolic-depolarization substrate at fixed EF

End-diastolic stretch was uniform across regions for uniform dilation but developed a septal-to-lateral gradient under asymmetric remodeling, with the thinned septum stretched most. The resulting substrate at severe asymmetric DCM was a 2.25 mV diastolic depolarization of the septum relative to baseline, 1.32 mV of regional resting-potential dispersion, and 2.19 ms of APD dispersion. Uniform dilation at the same EF produced 0.14 mV and 0.15 ms (**Figures 2**, **3B**). The substrate rose monotonically with asymmetry at every severity. The resting-potential and APD readouts tracked each other and both tracked wall stress heterogeneity (**Figure 3C**); these relationships are deterministic consequences of the coupled model and are reported as relationships and slopes rather than as correlations with significance values, which would be a category error for a deterministic model.

### 3.4 The substrate scales with an echo-computable gradient

**Figure 4:**
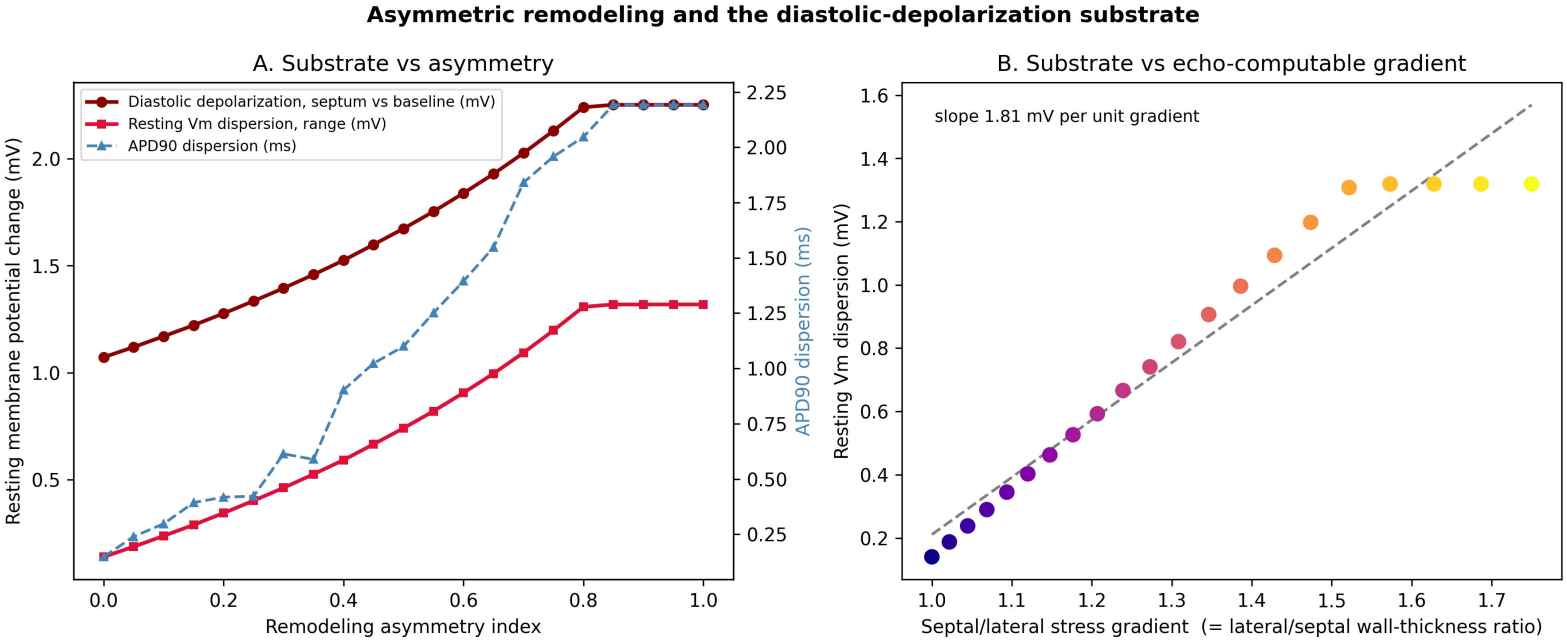
Substrate versus remodeling asymmetry and versus the echo-computable gradient, at severity 1.0. **(A)** Septal diastolic depolarization, resting-potential dispersion, and APD dispersion all rise with asymmetry and plateau once the septum reaches the sarcomere stretch ceiling. **(B)** Resting-potential dispersion versus the septal/lateral stress gradient, which equals the lateral-to-septal wall-thickness ratio; the relationship is approximately linear over the clinical range with a slope of 1.81 mV per unit gradient.

The substrate rose approximately linearly with the septal/lateral wall-stress gradient, which equals the lateral-to-septal wall-thickness ratio, at a slope of 1.81 mV of resting-potential dispersion per unit gradient over the clinically relevant range of gradients from 1.0 to roughly 1.5 (**Figure 4**). Above that range the relationship saturated as the septal stretch reached the sarcomere working-range ceiling and the SAC conductance saturated, a physical limit rather than a definitional artifact. There was no abrupt threshold; the relationship is continuous and then flat.

### 3.5 Single-cell validation and sensitivity

**Figure 5:**
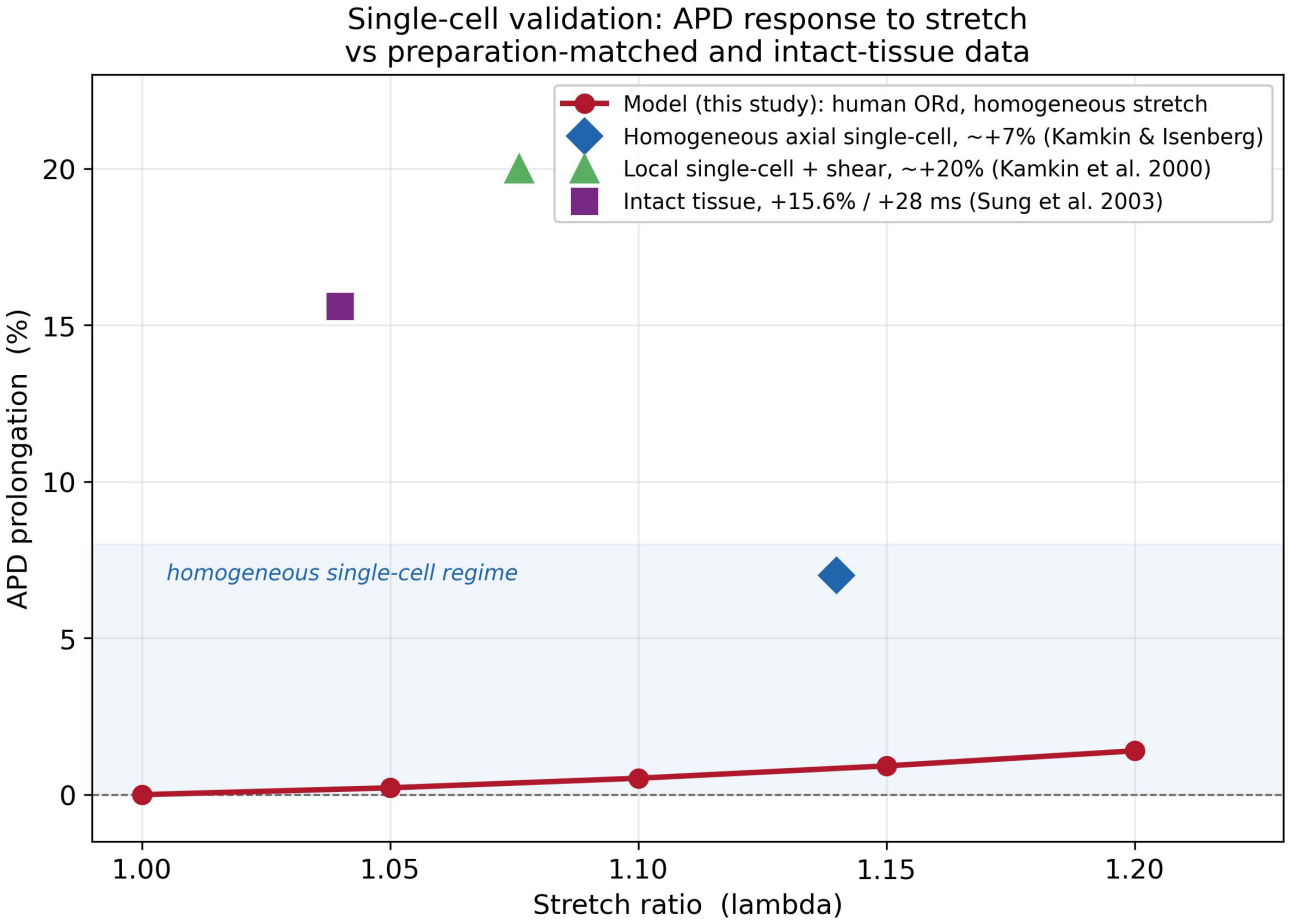
Single-cell validation: stretch-dependent APD prolongation. Model APD change (percent, prolongation positive) across the stretch range, against published experimental values grouped by preparation. The model corresponds to homogeneous single-cell stretch (shaded band), the preparation producing the smallest changes; local single-cell stretch with shear (Kamkin et al., 2000) and intact loaded tissue (Sung et al., 2003) show larger prolongation and are plotted for context at their reported stretch or strain. Stretch measure differs by preparation (sarcomere length versus epicardial strain). The prolongation direction reflects the chosen reversal potential and is preparation-dependent.

The SAC current produced a stretch-dependent APD90 prolongation of up to 3.2 ms, about 1.4%, at a stretch of 1.20, with a resting depolarization of up to 2.25 mV in the most-stretched region. The resting depolarization matches the roughly 2 mV reported for homogeneous axial single-cell stretch (Isenberg et al., 2003), and the APD change sits at the low end of the experimental range, consistent with that preparation; local-stretch and intact-tissue preparations show larger APD changes (**Figure 5**). This small per-cell magnitude is deliberate: the conductance rises only to a stretch of 1.2 and uses a conservative default maximum (Section 2.6). The central result does not rest on the single-cell magnitude but on the regional dispersion of resting potential and APD across the ventricle, which scales linearly with the conductance (Section 2.8) and is therefore preserved for any value in the published range.

The separation between asymmetric and uniform geometries was maintained across the published range of maximum SAC conductance, because the spatial stretch pattern is fixed by geometry and the conductance scales it monotonically, and was insensitive to the reversal potential, which scales the current equally across regions and leaves the relative dispersion unchanged.

## 4 Discussion

### 4.1 Principal finding and the nature of the claim

Asymmetric wall thinning, the pattern rather than the severity of DCM remodeling, produces a regional diastolic-depolarization and repolarization substrate that uniform dilation at the same EF does not. The substrate scales with the septal/lateral wall-thickness ratio and saturates at the sarcomere stretch ceiling. This is an in silico result, and it should be read as what such a model can legitimately provide: it does not discover a fact about real hearts but quantifies a hypothesized mechanism and produces a testable prediction. The chain from asymmetric thinning to stress heterogeneity to heterogeneous stretch to heterogeneous SAC engagement to dispersion of resting potential and repolarization is composed of individually established relationships, so the existence of the effect is expected. The contributions are its quantification, its orthogonality to EF, and its conversion into an echo-computable prediction.

### 4.2 Why the substrate is beyond ejection fraction

EF is a ratio of cavity volumes, and the wall-thickness distribution is not one of its inputs. The septal/lateral thickness ratio can vary while EF is unchanged, so the ratio carries information orthogonal to EF by the definition of EF, not merely beyond its sensitivity, and it is available on the same echocardiogram. Two ventricles with the same EF can therefore differ in the thickness-distribution-driven substrate (**Figure 2**). In the model EF is held independent of asymmetry by construction, through a fixed stroke volume and a cavity-defined EF; whether asymmetric remodeling independently depresses EF in patients, which would allow EF to capture part of the substrate, is an empirical question that this model cannot resolve and that retrospective clinical data should test.

### 4.3 Mechanistic interpretation

Each step has independent support. Regional diastolic wall-stress heterogeneity is documented in DCM (Hayashida et al., 1990). Stretch-activated channels in ventricular myocytes respond to diastolic lengthening with an inward depolarizing current (Kamkin et al., 2000; Peyronnet et al., 2016). Spatial dispersion of resting potential and repolarization is an established arrhythmogenic substrate in human ventricle (Glukhov et al., 2010). The primary readout used here, regional resting-potential dispersion, is the phase-appropriate consequence of diastolically engaged SACs and is itself a direct excitability substrate, because the resting potential sets sodium-channel availability and conduction. The direction of the APD change in the model is prolongation, consistent with non-selective-cation-dominant preparations; in other preparations stretch shortens APD. This directional ambiguity does not affect the central result, because the substrate is the regional gradient of repolarization and resting potential, not its sign.

### 4.4 Clinical translation

The septal/lateral gradient equals the lateral-to-septal wall-thickness ratio, computable from interventricular septal thickness and posterior or lateral wall thickness, both present on every standard echocardiogram. The model predicts that this ratio indexes a substrate that EF cannot encode. No specific cut-point is asserted, because the relationship is continuous and then saturating and because, without electrotonic coupling, the model produces a substrate proxy rather than a reentry threshold. The immediate validation step is retrospective: computing the gradient from existing echocardiographic databases with linked arrhythmia outcomes, such as MIMIC-IV-ECHO and the SHaRe DCM registry, and testing whether it adds predictive value over EF. Prospective validation would follow.

### 4.5 Limitations

First, the model has no electrotonic coupling, so it produces a substrate proxy and cannot claim reentry; for this reason no absolute-millisecond dispersion threshold is used, since the arrhythmogenic quantity is a steep local spatial gradient together with restitution and coupling rather than a fixed dispersion magnitude (Coronel et al., 2009; Laurita and Rosenbaum, 2000, reported a structure-dependent 3.2 ms per mm gradient, and Akar and Rosenbaum, 2003, a transmural gradient above 10 ms per mm). Second, thin-wall membrane stress diverges from finite-element fiber stress. Third, the SAC conductance set by diastolic stretch is applied as a constant through the beat rather than gated to the mechanical cycle. Fourth, the passive stress-to-strain relation is linearized and the reference is the loaded normal diastolic state rather than the unloaded configuration. Fifth, EF is held independent of asymmetry by a fixed stroke volume. Sixth, passive stiffness, diffuse interstitial fibrosis, and dilation etiology are not represented; fibrosis stiffens tissue regionally and would redistribute stretch (Wang et al., 2016), and volume-overload versus pressure-overload remodeling differ in their stress distribution (Grossman et al., 1975). Seventh, the model uses a single epicardial cell type and an idealized ellipsoid without explicit fiber architecture.

### 4.6 Future directions

The most direct next step is retrospective computation of the wall-thickness gradient from existing echocardiographic databases with arrhythmia outcomes. Computational extensions include a multicellular fiber model with electrotonic coupling to test whether the identified gradient produces reentrant circuits rather than dispersion alone (Trayanova, 2011; Niederer et al., 2019), and incorporation of fibrosis and ion-channel remodeling as concurrent substrates.

### 4.7 Conclusions

Asymmetric wall thinning, the pattern rather than the severity of DCM remodeling, produces a regional diastolic-depolarization and repolarization substrate that uniform dilation at the same ejection fraction does not. The substrate scales with the septal/lateral wall-thickness ratio, an echocardiographically computable quantity that is orthogonal to ejection fraction by definition. This in silico study quantifies the mechanism and yields a falsifiable prediction for retrospective and prospective clinical evaluation.

## Data Availability

All data reported in this study are generated by the computational model. Model code and generated data are available at https://github.com/arnava25/dcm-arrhythmia-model.
